# Supplementary material for: Harnessing the Dual Antimicrobial Mechanism of Action with Fe(8-Hydroxyquinoline)3 to Develop a Topical Ointment for Mupirocin-Resistant MRSA Infections
Source: Antibiotics (Basel). 2023 May 10;12(5):886. doi: 10.3390/antibiotics12050886 (PMC10215364; doi:10.3390/antibiotics12050886)
Supplement: Supplementary file 1 [file antibiotics-12-00886-s001.zip › antibiotics-2387563-supplementary.pdf]

Supporting Information

Harnessing the Dual Antimicrobial Mechanism of Action with Fe(8-Hydroxyquinoline)$_3$ to Develop a Topical Ointment for Mupirocin-Resistant MRSA Infections

Nalin Abeydeera [1], Bogdan M. Benin [2], Khalil Mudarmah [1,3], Bishnu D. Pant [1], Guanyu Chen [1], Woo Shik Shin [2,*], Min-Ho Kim [4] and Songping D. Huang [1,*]

[1] Department of Chemistry and Biochemistry, Kent State University, Kent, OH 44240, USA; nkekiriw@kent.edu (N.A.); kmudarma@kent.edu (K.M.); bpant@kent.edu (B.D.P.); gchen10@kent.edu (G.C.)
[2] Department of Pharmaceutical Sciences, College of Pharmacy, Northeast Ohio Medical University, Rootstown, OH 44272, USA; bbenin@neomed.edu
[3] Department of Chemistry, Jazan University, Jazan 45142, Saudi Arabia
[4] Department of Biological Sciences, Kent State University, Kent, OH 44240, USA; mkim15@kent.edu
* Correspondence: wshin@neomed.edu (W.S.S.); shuang1@kent.edu (S.D.H.)

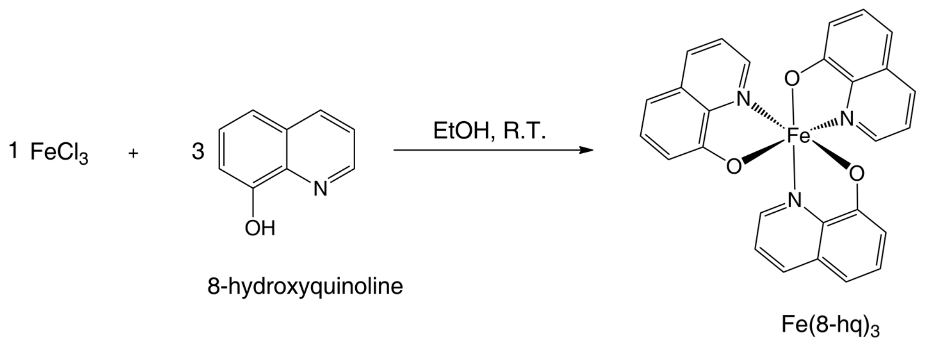

Scheme S1. Synthetic procedure for the preparation of Fe(8-hq)$_3$.

Characterization Studies

Table S1. Elemental analysis results of Fe(8-hq)$_3$.

Elements	$C_{27}H_{18}O_3N_3Fe$	
	Calculated %	Experimental %
Carbon (C)	66.41	66.11
Hydrogen (H)	3.72	3.71
Nitrogen (N)	8.61	8.73
Iron (Fe)	11.44	11.52

Empirical formula related to experimental values of the Fe(8-hq)$_3$ complex gives the exact molecular formula and experimental values are closer to the calculated values. This results undoubtedly confirmed the identity of the obtained product to be Fe(8-hq)$_3$ with purity >98%.

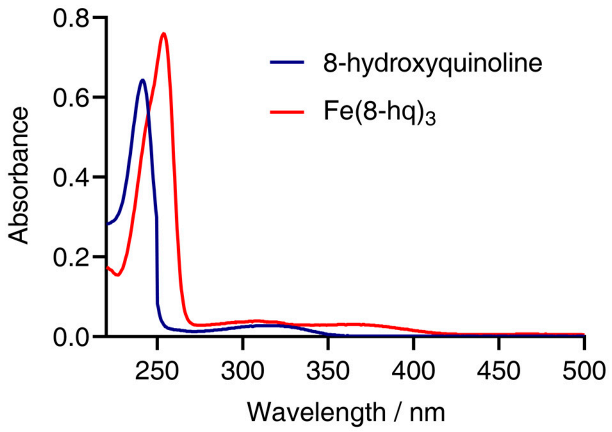

Figure S1. UV-vis spectra of Fe(8-hq)$_3$ and 8-hydroxyquinoline in chloroform.

UV-visible spectra of 8-hydroxyquinoline and Fe(8-hq)3 were recorded in chloroform showed significant differences between the absorption peaks of 8-hydroxyquinoline and Fe(8-hq)3 complex. Since ligand bear conjugated systems, π–π* transition is possible. In Fe(8-hq)3 complex, the wavelengths have shifted towards the longer wavelength range (bathochromic shift) because of changes in the conjugated electron system due to the formation of metal-ligand bonds.

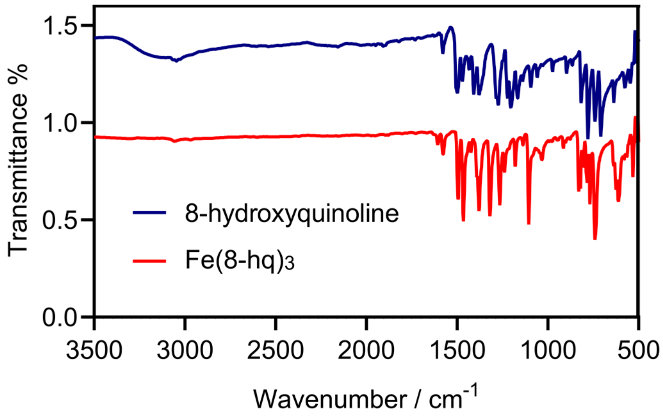

Figure S2. FT-IR spectra of Fe(8-hq)3 and 8-hydroxyquinoline.

The dried metal complex product and ligand were produced, and FT-IR data was acquired. The stretching frequency of the 8-hydroxyquinoline ring (C-N) and the stretching frequency of the 8-hydroxyquinoline ring (C-O) are crucial. These stretching frequencies have changed because of the creation of new metal-ligand connections. Therefore, by measuring the stretching frequency of the same bond, we may determine whether the reaction has occurred. In addition, because of the development of new metal-ligand bonds, the stretching frequency of the C-N and C-O bonds has been changed to a lower frequency range, lowering their strength.

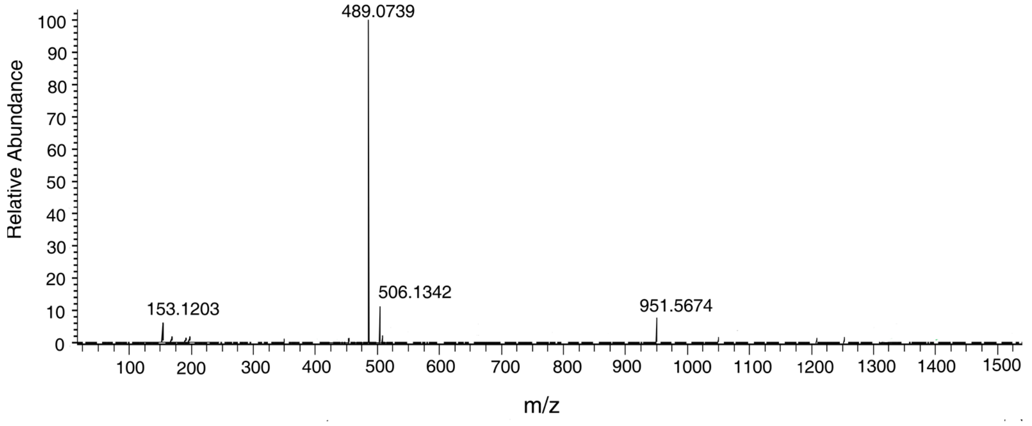

Figure S3. ESI/LC-MS trace of the as-synthesized Fe(8-hq)3.

ESI/LC-Mass spectrometry was used to identify the structure and purity of the synthesis product. We observed the appearance of a signal (m/z of 489.073) which corresponds to Fe(8-hq)3 and clearly confirmed the identity of the obtained product to be Fe(8-hq)3.

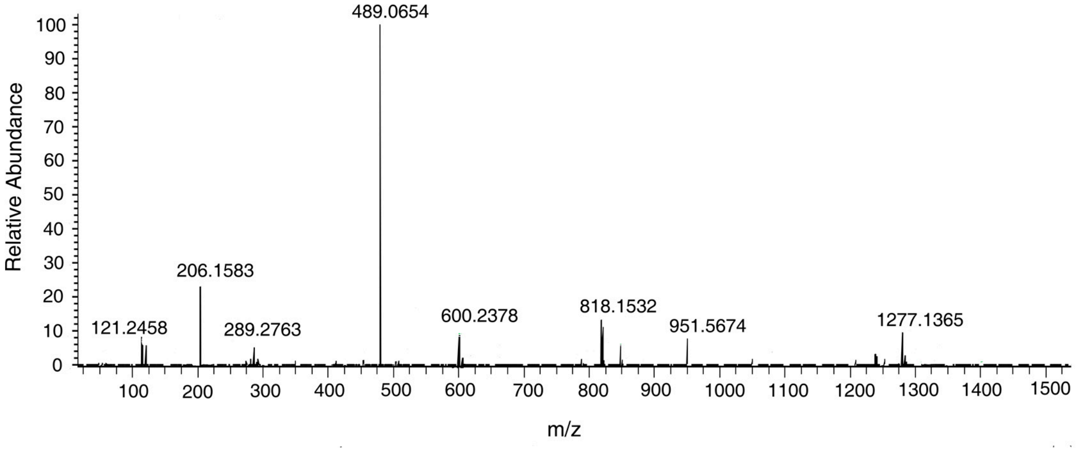

Figure S4. ESI/LC-MS trace of the recovered product Fe(8-hq)3 after incubating at 37 ºC and 180 rpm for 48 hours with the bacterial cell culture medium. When compared to the results shown in **Figure S3**, it confirms the stability of Fe(8-hq)3 against the degradation in the cell culture medium or the formation of protein aggregates.

Biological Assays

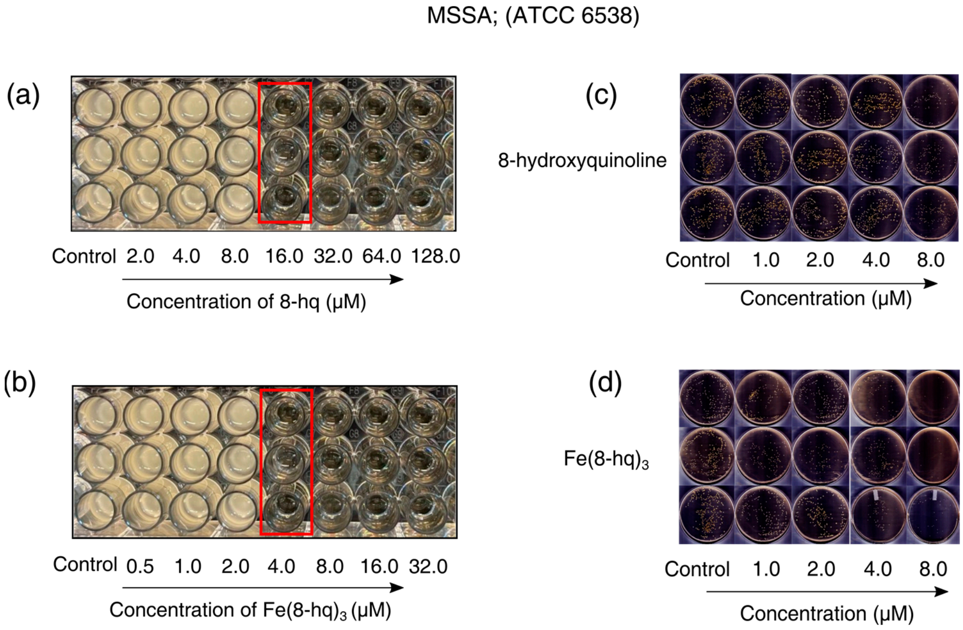

Figure S5. Representative images of MIC test of 8-hydroxyquinoline (**a**), Fe(8-hq)3 (**b**) and representative images of CFU enumeration results (**c** and **d**) against MSSA (ATCC 6538).

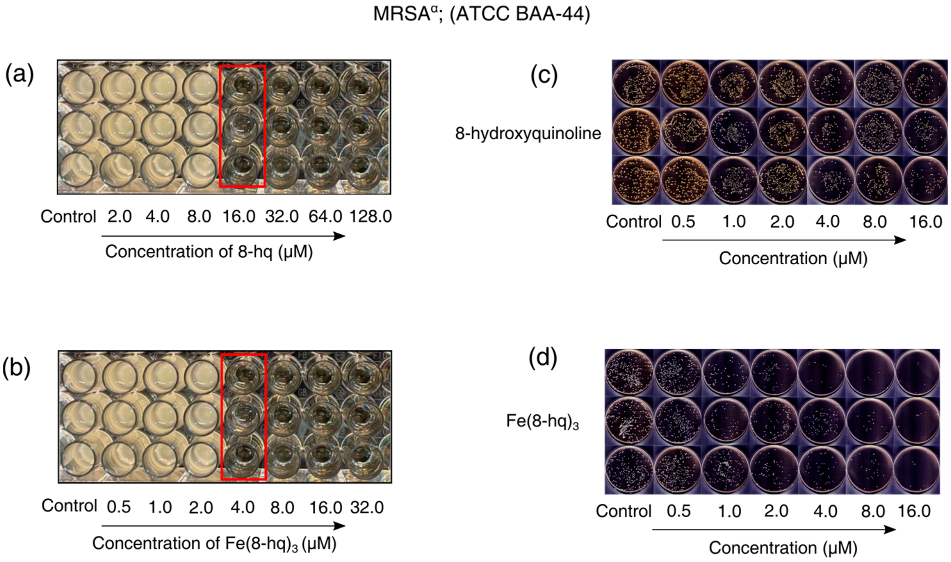

Figure S6. Representative images of MIC test of 8-hydroxyquinoline (**a**), Fe(8-hq)3 (**b**) and representative images of CFU enumeration results (**c** and **d**) against MRSA$^{\alpha}$ (ATCC BAA-44).

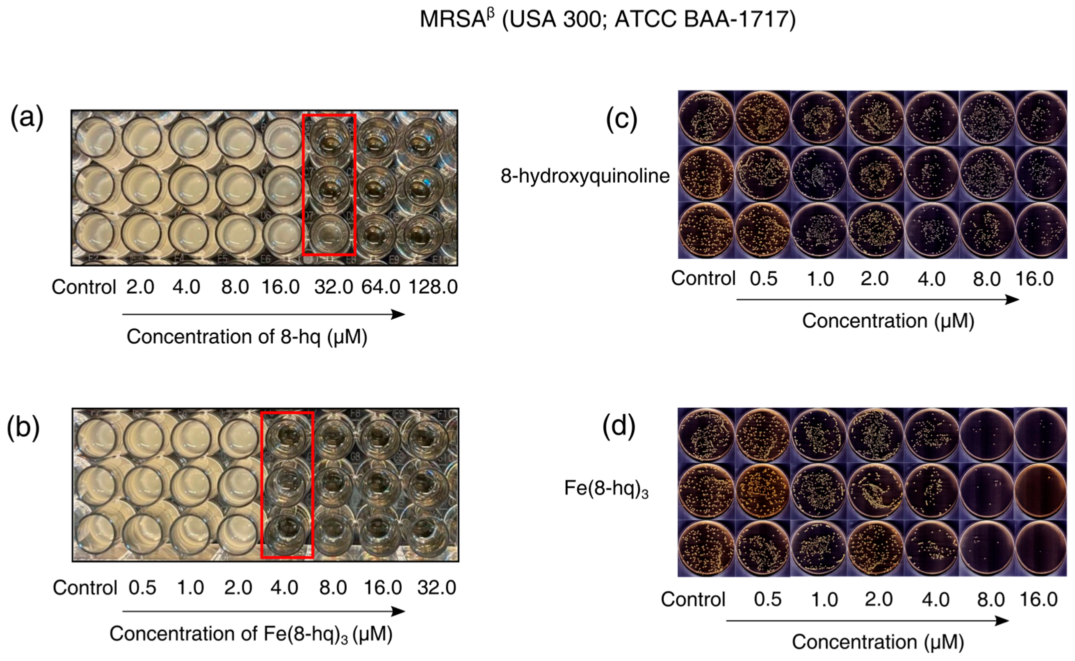

Figure S7. Representative images of MIC test of 8-hydroxyquinoline (**a**), $Fe(8\text{-}hq)_3$ (**b**) and representative images of CFU enumeration results (**c** and **d**) against $MRSA^{\beta}$ (USA 300; ATCC BAA-1717).

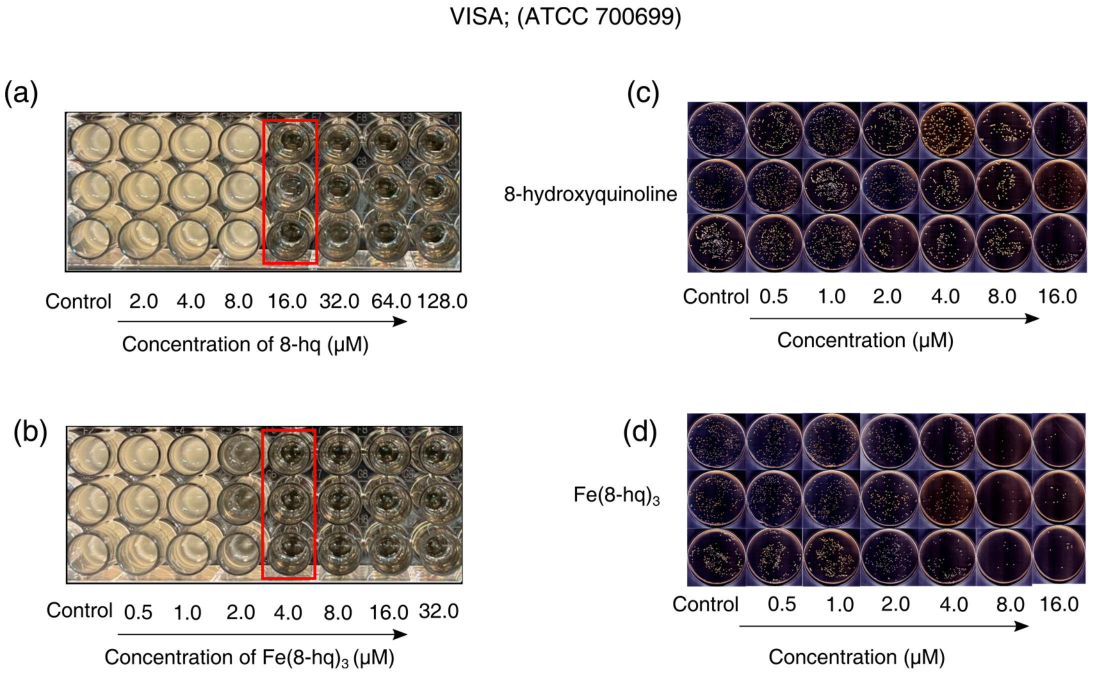

Figure S8. Representative images of MIC test of 8-hydroxyquinoline (**a**), $Fe(8\text{-}hq)_3$ (**b**) and representative images of CFU enumeration results (**c** and **d**) against VISA (ATCC 700699).

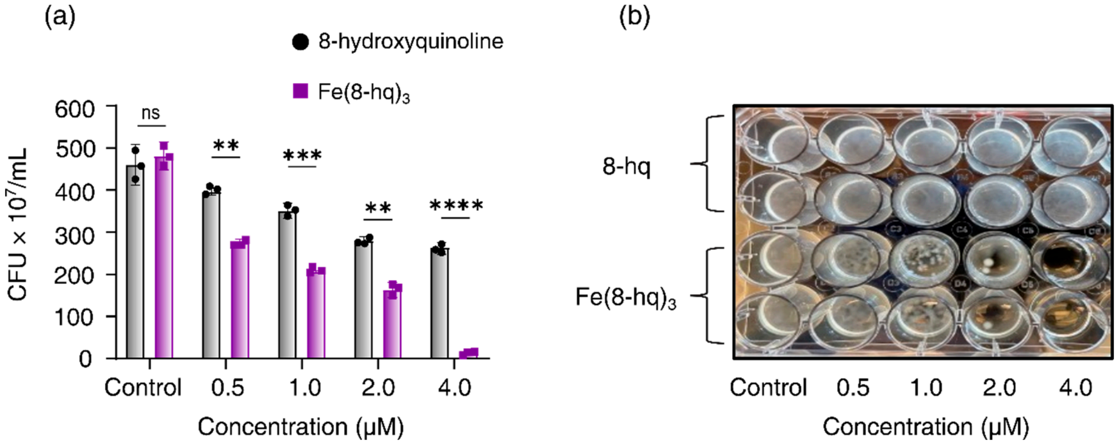

Figure S9. Growth-inhibitory effect of $Fe(8\text{-}hq)_3$ in comparison with molar equivalent concentrations of 8-hq in biofilm-derived MRSA[α] bacterial cells (**a**) and representative images of biofilm-inhibitory results (**b**) (mean ± s.d, n =3 replicates; *$p < 0.05$, **$p < 0.01$, ***$p < 0.001$, ****$p < 0.0001$, ns = not significant).

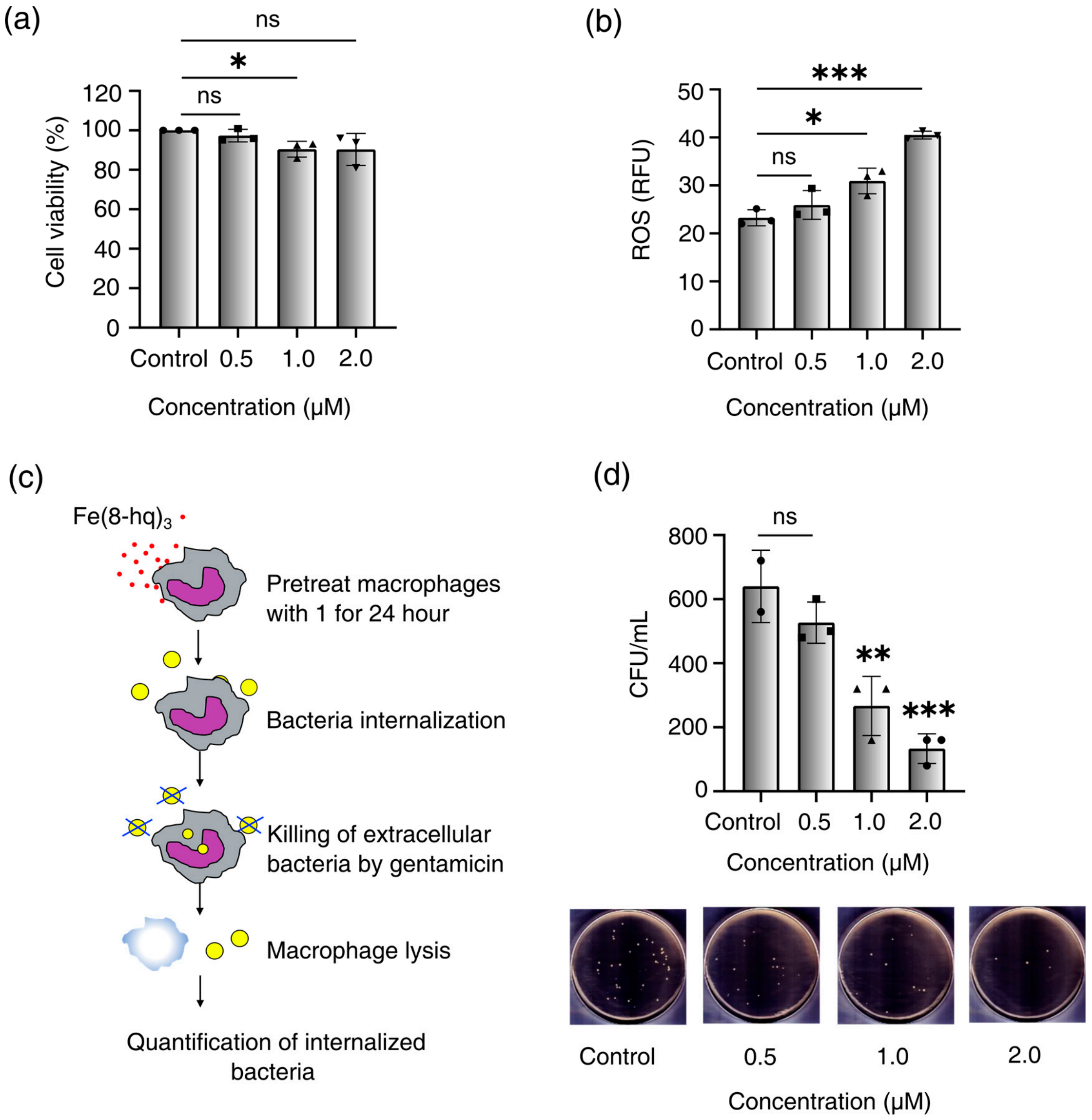

Figure S10. The effect of Fe(8-hq)$_3$ on the viability of RAW 264.7 cells treated with varying concentrations between 0 and 2 μM for 24 h (**a**). The quantification of ROS generation in RAW 264.7 cells treated with varying concentration between 0 and 2 μM for 18 h (**b**). A schematic diagram of the experimental protocol for phagocytosis and killing of intracellular bacteria by an antibiotic protection assay (**c**). The effect of varying concentrations of Fe(8-hq)$_3$ between 0 and 2 μM on the bactericidal activity of RAW 264.7 cells assessed by the antibiotic protection assay (**d**) (mean ± s.d, n =3 replicates; *p < 0.05, **p < 0.01, ***p < 0.001 and ns = not significant).

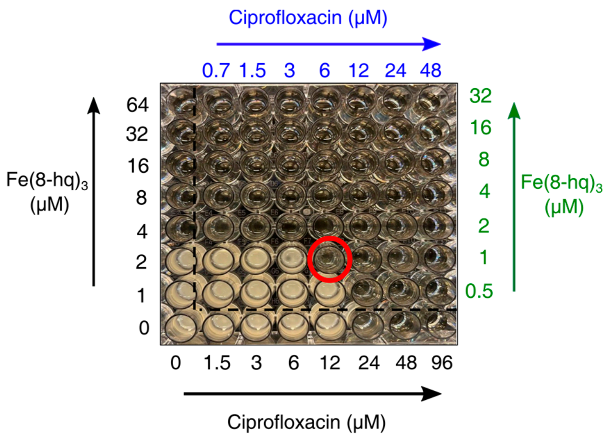

Figure S11. Checkerboard assay for Fe(8-hq)$_3$ and ciprofloxacin against MRSA$^\alpha$ with a representative image of MIC assay. The green arrow/font and blue arrow/font indicate the concentration of Fe(8-hq)$_3$ and ciprofloxacin, respectively, used for the determination of MIC for the combination of ciprofloxacin and Fe(8-hq)$_3$, where the MIC was determined to be of 6.0 μM of ciprofloxacin in the presence of 1.0 μM of Fe(8-hq)$_3$ (red circled-well).

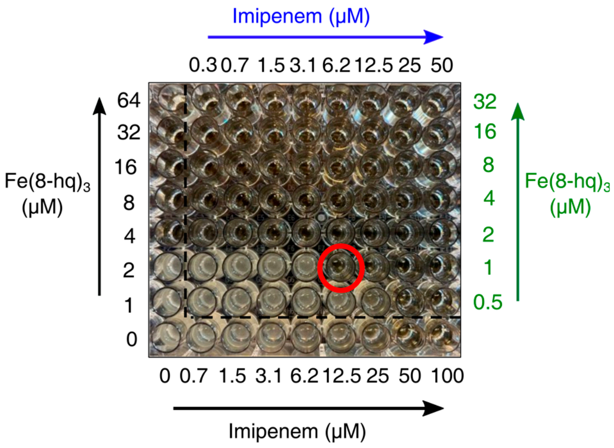

Figure S12. Checkerboard assay for Fe(8-hq)$_3$ and imipenem against MRSA$^\alpha$ with a representative image of MIC assay. The green arrow/font and blue arrow/font indicate the concentration of Fe(8-hq)$_3$ and imipenem, respectively, used for the determination of MIC for the combination of imipenem and Fe(8-hq)$_3$, where the MIC was determined to be of 6.25 μM of imipenem in the presence of 1.0 μM of Fe(8-hq)$_3$ (red circled-well).

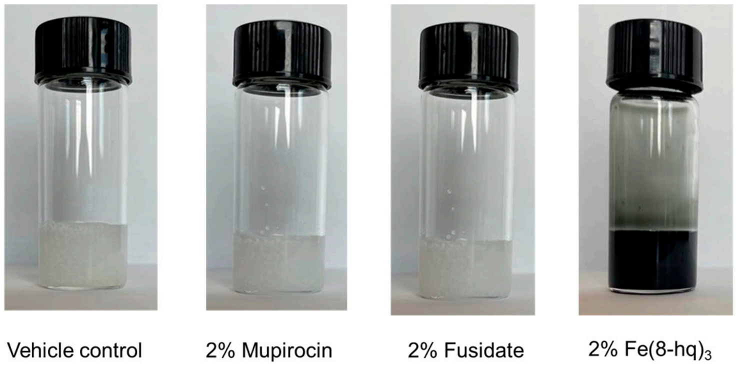

Figure S13. Representative images of prepared PEG-based vehicle control, 2% mupirocin, 2% fusidate and 2% $Fe(8\text{-}hq)_3$.

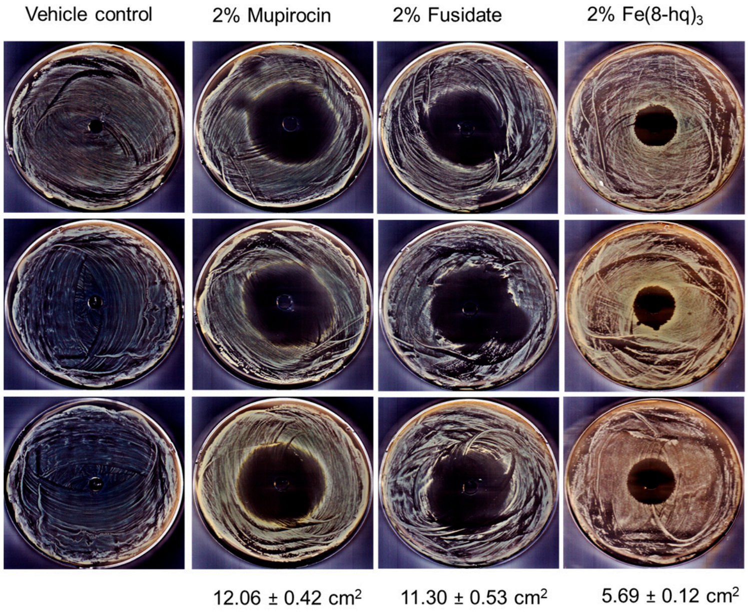

Figure S14. Antimicrobial effects of PEG-based ointments containing vehicle control, 2% mupirocin, 2% fusidate, and 2% $Fe(8\text{-}hq)_3$ toward wild type MRSA$^{\alpha}$.

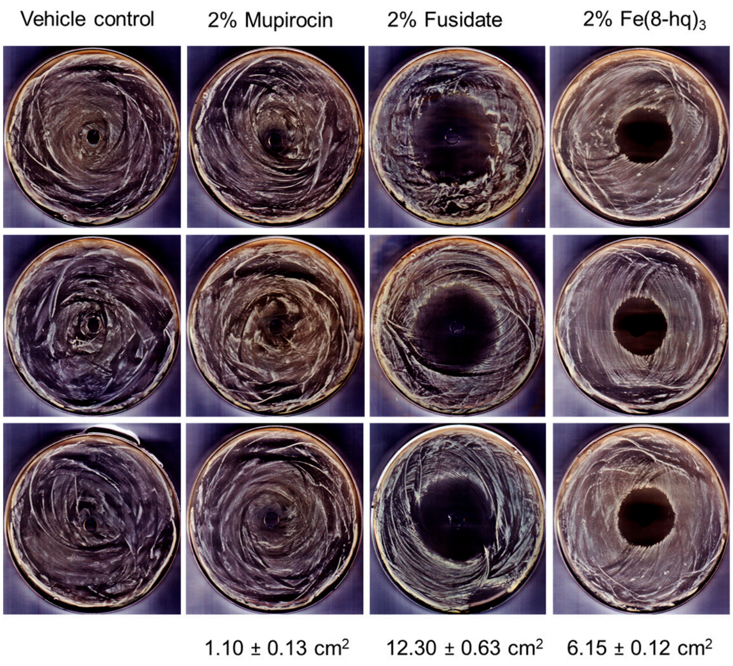

Figure S15. Antimicrobial effects of PEG-based ointments containing vehicle control, 2% mupirocin, 2% fusidate, and of 2% $Fe(8\text{-}hq)_3$ toward high-level mupirocin resistant MRSA$^{\alpha}$; MRSA$^{(mupR)}$.

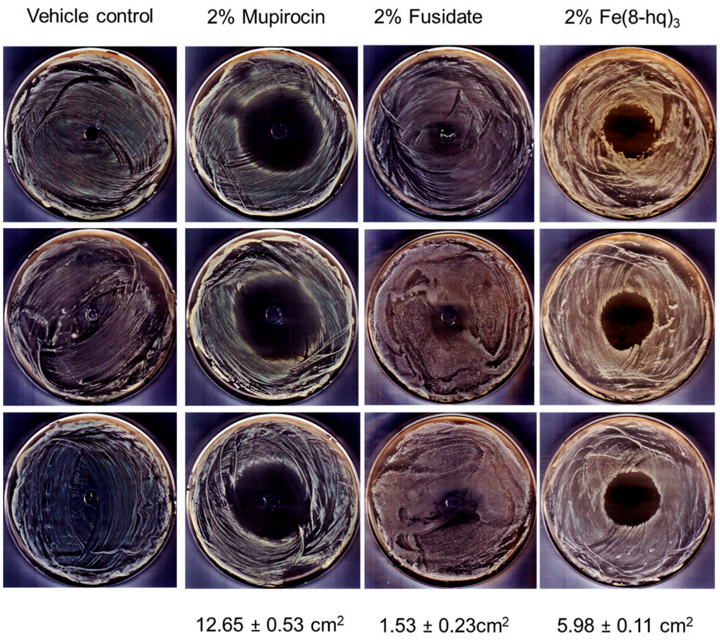

Figure S16. Antimicrobial effects of PEG based ointments containing vehicle control, 2% mupirocin, 2% fusidate, and of 2% $Fe(8\text{-}hq)_3$ toward high-level fusidate-resistant MRSA$^{\alpha}$; MRSA$^{(fusR)}$.

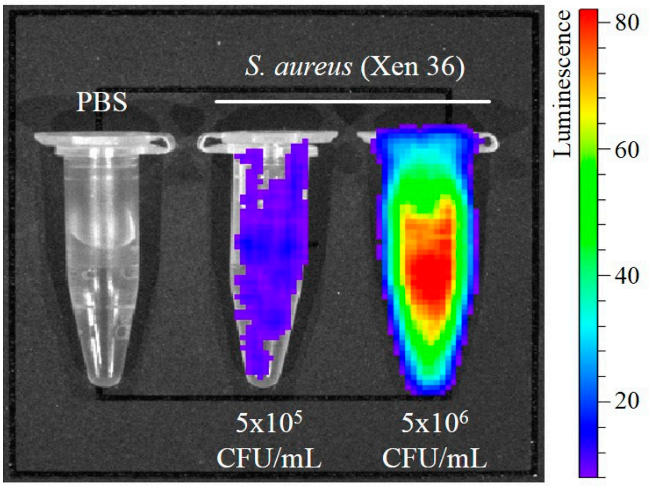

Figure S17. IVIS imaging of *S. aureus* (Xen 36) for the number of CFU optimization before mice infection.

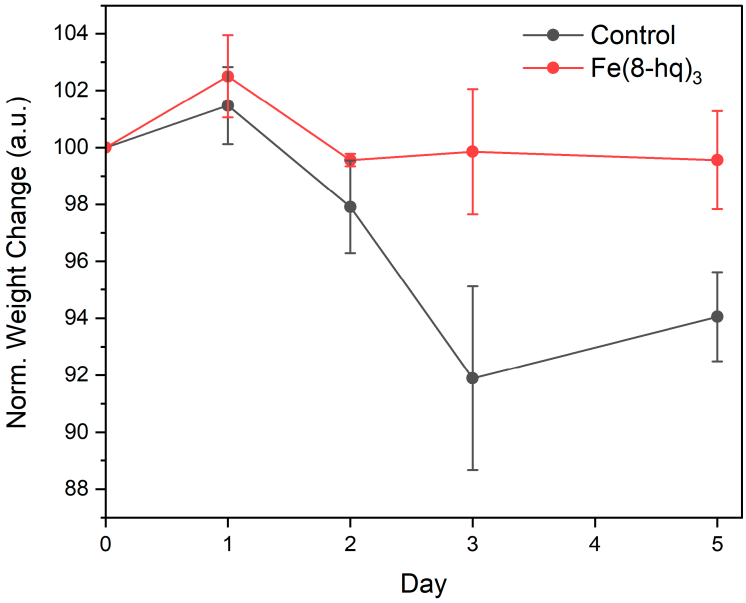

Figure S18. The group averaged mouse weight following either vehicle control or $Fe(8\text{-}hq)_3$ treatment.
